# Infection prevention and control and related practices in African neonatal units: The Pan-African neonatal care assessment study (PANCAS)

**DOI:** 10.1016/j.ijheh.2024.114357

**Published:** 2024-06

**Authors:** Irene Frantzis, Stéphanie Levasseur, Jack Huebner, Maitry Mahida, Philip Larussa, Wilmot James, Workeabeba Abebe, Crispen Ngwenya, Ezekiel Mupere, Susan L. Rosenthal, Janna Patterson, Julia Johnson, Renate Strehlau, Sileshi Lulseged, Lawrence R. Stanberry, Lisa Saiman

**Affiliations:** a Department of Pediatrics, Columbia University Irving Medical Center, New York, New York, USA; b Department of Health Services, Policy and Practice, School of Public Health, Brown University, Providence, RI, USA; c Tikur Anbessa Specialized Hospital, Addis Ababa University, Addis Ababa, Ethiopia; d Paediatrics department at Midlands State University faculty of Medicine, Gweru, Zimbabwe; e Department of Paediatrics and Child Health School of Medicine College of Health Sciences, Makerere University, Kampala, Uganda; f Departments of Pediatrics and Psychiatry, Columbia University Vagelos College of Physicians and Surgeons, New York, New York, USA; g Global Child Health and Life Support, American Academia of Pediatrics, Itasca, IL, USA; h Division of Neonatology, Department of Pediatrics, Johns Hopkins University School of Medicine, Baltimore, MD, USA; i VIDA Nkanyezi Research Unit, Department of Paediatrics and Child Health, Rahima Moosa Mother and Child Hospital, Faculty of Health Sciences, University of the Witwatersrand, Johannesburg, South Africa; j Department of Pediatrics and Child Health, College of Health Sciences, Addis Abbaba, Ethiopia; k Department of Infection Prevention and Control, NewYork-Presbyterian Hospital, New York, NY, USA

**Keywords:** Africa, Global health, Neonates, Newborns, Infection prevention

## Abstract

**Background:**

The burden of neonatal mortality is primarily borne by low- and middle-income countries (LMICs), including deaths due to healthcare-associated infections (HAIs). Few studies have assessed infection prevention and control (IP&C) practices in African units caring for small and/or sick newborns aimed to reduce HAIs.

**Methods:**

We performed a mixed-methods study composed of a survey and virtual tour to assess IP&C and related practices. We created a survey composed of multiple-choice and open-ended questions delivered to site respondents via Zoom or video equivalent. Respondents provided a virtual tour of their unit via video and the study team used a checklist to evaluate specific practices.

**Results:**

We recruited 45 units caring for small and sick newborns in 20 African countries. Opportunities to optimize hand hygiene, Water, Sanitation and Hygiene (WASH) practices, Kangaroo Mother Care, and IP&C training were noted. The virtual tour offered further understanding of IP&C challenges unique to individual sites. All respondents expressed the need for additional space, equipment, supplies, education, and IP&C staff and emphasized that attention to maternal comfort was important to IP&C success.

**Discussion:**

This study identified opportunities to improve IP&C practices using low-cost measures including further education and peer support through learning collaboratives. Virtual tours can be used to provide site-specific assessment and feedback from peers, IP&C specialists and environmental engineering experts.

## Abbreviations

HAI: Healthcare associated infections
LMIC: Low- and middle-income countries
IP&C: Infection prevention and control
WASH: Water sanitation and hygiene
KMC: Kangaroo mother care
MDRO: Multi-drug resistant organisms

## 1 Introduction

The burden of neonatal mortality is primarily borne by low- and middle-income countries (LMICs) where the risk of death in newborns is 10–15 times higher than in high-income countries (HIC) (Wang et al., 2016). While most deaths are attributed to delivery complications or early onset sepsis, deaths due to potentially preventable healthcare-associated infections (HAIs) in hospitalized small and/or sick neonates are also of increasing concern, (Olivier et al., 2018) particularly those caused by multidrug-resistant organisms (MDROs), requiring treatment with costly, broad-spectrum antibiotics (Morkel et al., 2014).

In May 2022, the World Health Organization (WHO) attributed adverse impacts on human health to lack of infection prevention and control (IP&C) strategies in LMICs, including suboptimal water supplies, sanitation, hygiene, and waste management (WASH) services (World Health Organization, 2022). The WHO recommended local, national, and global priorities (including political commitment); policies to develop and sustain IP&C programs; training and continuous education to strengthen the specialty of IP&C; and surveillance systems to monitor HAIs, emerging pathogens, and WASH practices.

In LMICs and HIC, improving hand hygiene, environmental and equipment cleaning, and policies to decrease crowding have reduced HAIs in newborns (Harris et al., 2019; Seale et al., 2009; Conde-Agudelo and Díaz-Rossello, 2014; Cross et al., 2019; Gon et al., 2021). Furthermore, in LMICs, Kangaroo Mother Care (KMC) has significantly reduced healthcare-associated sepsis, and thus serves as an IP&C strategy (Arya et al., 2023). Yet, few studies have systematically evaluated IP&C practices in African hospitals caring for small and/or sick neonates (Harris et al., 2019; Cross et al., 2019; Asare et al., 2009; Dramowski et al., 2016, 2021). Thus, the objectives of this study were to [1] assess specific IP&C practices, including KMC, in African hospitals caring for small and/or sick newborns <30 days old, [2] identify gaps in IP&C practices to inform future strategies to improve these practices, and [3] describe the needs and priorities of clinicians caring for this population.

## 2 Methods

### 2.1 Study design, site recruitment, survey respondents

To assess IP&C practices in diverse hospitals in sub-Saharan Africa caring for small and/or sick newborns, we developed a mixed-methods study consisting of a survey complemented by a virtual assessment of the care units and by clinicians’ self-reported needs and priorities for newborn care.

Potential sites were identified from past partnerships, referrals from global health physicians, or snowball referrals from participating sites. Sites received an email invitation (English or French) that provided an overview of the study's purpose and survey content. Three attempts to contact each site were made. Each individual site selected a respondent(s) familiar with IP&C practices, e.g., unit medical director, to answer the survey and/or conduct the video tour. The respondents were encouraged to consult other team members (e.g., the charge nurse, environmental workers) for assistance completing the survey. The Columbia University Irving Medical Center (CUIMC) team indicated particular sections of the survey for which multidisciplinary team input might be needed.

Approval from the CUIMC Institutional Review Board was obtained. Sites obtained local ethics approval as per local requirements. Sites received $1000 USD to defray the costs associated with this research, e.g., IRB fees.

### 2.2 Survey development

The CUIMC study team, comprised of experts in global health and IP&C, developed a survey based on guidelines for evidence-based IP&C practices for units caring for small and/or sick newborns (Infection Prevention and control Assessment, 2018). The survey assessed multiple domains including space and unit layout, visitor policies, KMC, systems capacity, environmental and equipment cleaning, WASH practices, and available supplies (Supplement 1). In addition, the survey asked about hospital and patient characteristics and the impact of the COVID-19 pandemic. Two open-ended questions investigated respondents’ greatest needs, i.e., what they would fund if money was available.

The draft survey was reviewed by physicians from different African hospitals, physicians with global health experience from the American Academy of Pediatrics Global Child Health and Life Support section, and the Bill and Melinda Gates Foundation, and was modified accordingly. CUIMC public health students, infection preventionists, and pediatricians with experience in neonatal care pilot-tested the survey resulting in additional modifications. The final survey was translated into French.

The survey used Yes/No/Unsure responses, multiple choice questions, and open-ended questions. In multiple-choice questions, the option of “other” was offered to allow additional options that were then incorporated into the survey. To reduce recall bias for specific items, e.g., average daily census or availability of certain supplies, respondents provided responses based on the day the survey was administered and were asked if “this was a typical day?”

### 2.3 Virtual assessments

To complement the survey, we developed a virtual assessment tool and conducted a video tour via Zoom or WhatsApp on the same day as the survey, whenever feasible. Areas to assess were selected based on team members’ prior site visits to pediatric hospital wards across Sub Saharan Africa (Tarun et al., 2023) (e.g., patient and visitor crowding, KMC capacity, sink accessibility), as well as informed by IP&C observational studies conducted in the U.S. and other countries (e.g., hand hygiene supplies, rubbish bin accessibility, sharps safety). IP&C observations that would identify patients, staff, or visitors were avoided. Thus, during the tour, respondents provided images of the items on the virtual assessment checklist avoiding patient, visitor, or staff faces or other identifiers. Respondents were asked to clarify images if something was not easily visible, e.g., presence of window screens or running water. If a specific area was unavailable for observations, it was not scored. Alternatives e.g., a video or photographs via encrypted messaging, were arranged if internet connectivity was suboptimal. Given the potentially subjective nature of the items scores (e.g., crowded conditions), at least two team members independently assigned a score and then discussed the results. To ensure further consistency of interpretation, one team member (IF) reviewed recordings of each virtual assessment. Discrepancies were resolved by adjudication from the rest of the team.

### 2.4 Analysis

Data were entered into REDCap for analysis. Observations of each item on the virtual assessment were scored 0–2 by two members of the CUIMC study team; higher scores reflected more favorable IP&C observations. Discrepancies were resolved by consensus with the study team. Descriptive statistics (frequencies, means, medians, ranges) were performed for survey responses and virtual observations. Responses to open-ended questions were transcribed; French responses were translated to English. The transcripts were reviewed to develop themes used to analyze responses to both questions. Transcripts were coded independently by two team members. Coded themes were not mutually exclusive. Discrepancies were resolved through consensus.

## 3 Results

### 3.1 Hospitals characteristics

**Fig. 1:**
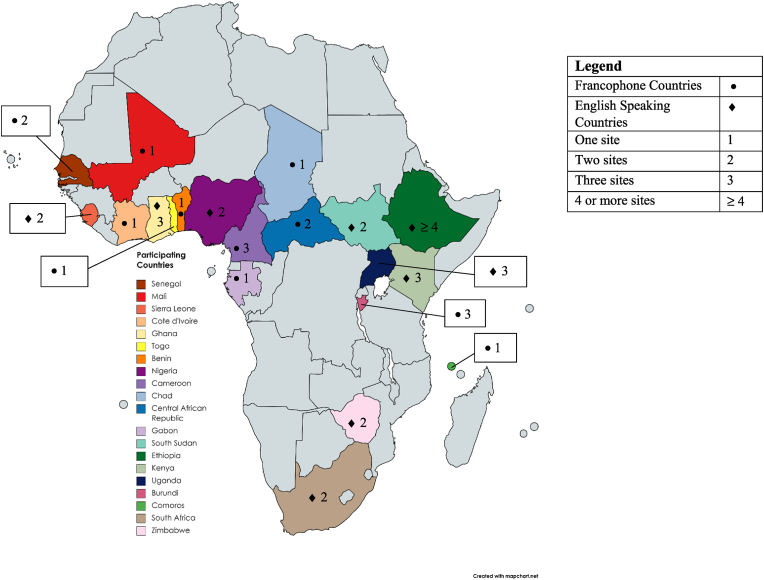
Participating countries and number of sites.

Ninety-six hospitals from 33 countries were contacted of which 45 (47%) participated from 20 countries (Fig. 1). Twenty-one (46%) hospitals were academically affiliated. Thirty were national/regional and 8 were district/provincial hospitals; 7 did not report these classifications. Forty (89%) had public funding, Two each reported being privately funded or funded by Missionary services, and one hospital was unsure about funding sources.

Infrastructure of the units that cared for small and/or sick newborns is shown (Table 1). Most (67%) had neonatal ICUs and a KMC room. Fewer had units to separate preterm and term newborns or an isolation area. Six hospitals had a single room for all newborns, and one had no designated area. All hospitals accepted newborns born at home or transferred from another hospital, and 33 (73%) readmitted newborns who had been discharged.

**Table 1:** Infrastructure of sites providing care for small and/or sick newborns <30 days old.

Infrastructure of newborn unit	All sites N = 45	Academic^a^ n = 21	Non-academic^a^ n = 24
Neonatal intensive care unit	24 (53%)	14 (67%)	10 (42%)
Kangaroo Mother Care room	24 (53%)	14 (67%)	10 (42%)
Separate preterm/term units	18 (40%)	10 (47%)	8 (33%)
Separate inborn/out-born units	15 (33%)	8 (38%)	7 (29%)
Observation/step-down unit	13 (29%)	10 (47%)	3 (12%)
Isolation area	7 (15%)	5 (24%)	2 (8%)
Single neonatal room only	6 (13%)	1 (5%)	5 (21%)
No designated neonatal area	1 (2%)	0	1 (4%)

a Self-designated.

On the survey day, the mean census was 28 (median 19, range 0–95) small and/or sick newborns. On average, 9 newborns (median 5, range 0–41) had a birthweight <1500 g. Ten (22%) sites reported their census was usually higher than the survey day. A median of 10 (range 3–50) nurses, and 5 (range 1–20) physicians were working throughout the 24-h period of the survey day. The median physician-to-patient ratio was 1:5 (maximum 1:34). All reported that this staffing reflected a typical day. Forty (89%) hospitals employed a physician(s) who completed pediatric training including 19 (42%) that employed a physician(s) who also completed neonatology training.

Extended family members and children were permitted to visit at 4 (9%) and 5 (11%) hospitals, respectively. Twenty-one (48%) hospitals reported two or more newborns (not including twins) were sharing isolettes/cribs. Thirty-five sites (78%) reported this was a typical day. The virtual assessment (Table 2) confirmed crowding with crib sharing (40%), and cribs touching (46%). Additional observations included visitors impeding workflow (20%), and crowded KMC areas (52%).

**Table 2:** Virtual tour assessment checklist results.

Virtual assessments	Visualized element	Observations n (%)
Crib sharing	**0:** ≥ half of cribs are holding more than one newborn	5 (11%)
	**1:** fewer than half of cribs holding more than one newborn	13 (29%)
	**2:** no newborns sharing cribs	26 (58%)
	**N/A:** unable to assess	1 (2%)
Crib spacing	**0:** most cribs touching	5 (11%)
	**1:** some cribs touching	16 (35%)
	**2:** no cribs touching	24 (53%)
	**N/A:** unable to assess	0 (0%)
Bedside area	**0:** obscured with clutter potentially unrelated to patient care	6 (13%)
	**1:** some potentially removable clutter	14 (31%)
	**2:** no clutter around bedside area	22 (49%)
	**N/A:** unable to assess	3 (7%)
Visitors	**0:** visitors/belongings impeding staff work/flow	3 (7%)
	**1:** visitors potentially impeding staff work/flow	6 (13%)
	**2:** visitors not impeding staff work/flow	35 (78%)
	**N/A:** unable to assess	1 (2%)
Kangaroo care	**0:** no newborns are receiving Kangaroo care	5 (11%)
	**1:** newborns observed receiving Kangaroo care in crowded conditions	23 (52%)
	**2:** newborns observed receiving Kangaroo care in uncrowded conditions	11 (25%)
	**N/A:** unable to assess	5 (11%)
Access to sinks	**0:** no access to sinks/multiple barriers	8 (18%)
	**1:** difficult to access sinks due to potential barriers	11 (24%)
	**2:** easy access to sinks with minimal barriers	26 (58%)
	**N/A:** unable to assess	0 (0%)
Ventilation	**0:** more than half of windows are open	7 (16%)
	**1:** less than half of windows are open	15 (33%)
	**2:** No open windows	22 (49%)
	**N/A:** unable to assess	1 (2%)
Screens	**0:** no screens on windows	16 (36%)
	**1:** some windows have screens	8 (18%)
	**2:** all windows have screens	13 (29%)
	**N/A:** unable to assess	8 (18%)
Sharps containers	**0:** no sharps containers accessible	2 (4%)
	**1:** sharps container accessible, but overflowing	9 (20%)
	**2:** sharps container accessible, not overflowing	34 (76%)
	**N/A:** unable to assess	0 (0%)
Sharps container location	0: sharps containers on floor	24 (53%)
	**1:** sharps containers on counter/bedside and not secured	14 (31%)
	**2:** sharps containers secured to wall	6 (13%)
	**N/A:** unable to assess	1 (2%)
Rubbish cans	**0:** no rubbish cans accessible	4 (9%)
	**1:** rubbish cans accessible, but overflowing	4 (9%)
	**2:** rubbish cans accessible, not overflowing	36 (80%)
	**N/A:** unable to assess	1 (2%)
Procedure area	**0:** significant uncleanliness compromising to performed procedures	2 (4%)
	**1:** potential uncleanliness compromising to performed procedures	13 (29%)
	**2:** no uncleanliness	16 (35%)
	**N/A:** unable to assess	14 (31%)
Hand hygiene/sink	***Assign 1 point for each supply***	3 supplies, n = 7 (16%)
	1- Disposable towel	2 supplies, n = 24 (53%)
	1- Soap or hand sanitizer	1 supply, n = 10 (22%)
	1- Running water	0 supplies, n = 4 (9%)

### 3.2 Kangaroo Mother Care

KMC was available in 42 (93%) sites. Nine (21%) were able to provide KMC at the newborn's bedside, 26 (62%) emphasized starting KMC as soon as possible, and 12 (29%) provided intermittent KMC to critically ill newborns. On the survey day, 23% (mean per site, range 0–100%) of newborns were receiving KMC which 76% of sites reported was a typical day.

All respondents reported their staff believed in KMC benefits, 37 (82%) reported that most mothers believed in KMC benefits, and 26 (62%) reported families supported mothers to perform KMC. Sixteen (38%) respondents reported their hospital was unable to optimally support KMC, primarily due to staffing shortages (n = 12/16, 75%). Respondents reported that 6 (mean per site, range 0–30) additional newborns could receive KMC if more resources were available. The most common challenges to providing KMC were family support/cultural incompatibility (40/45, 89%) and maternal and newborn medical conditions (69% and 61%, respectively). The most common support needed to increase KMC was educational material and training (35/43, 78%). Additional challenges and resources needed for KMC are shown (Table 3).

**Table 3:** Available resources, challenges and support needed to optimize KMC.^a^

**Maternal resources (n = 42)**	

Beds	36 (86%)
Restrooms	33 (79%)
Water	28 (67%)
Food	21 (50%)
**Educational resources (n = 42)**	
*For staff*	
Lectures/demonstrations	22 (49%)
Guidelines	16 (35%)
Posters	16 (35%)
*For mothers*	
Posters	13 (45%)
Bedside Educations	12 (41%)
Pamphlets	6 (21%)
Videos	4 (14%)
KMC Champions	4 (14%)
**Challenges providing KMC (n = 45)**	
Family support/Cultural incompatibility	40 (89%)
Maternal medical condition	31 (69%)
Newborn medical condition	28 (62%)
Adequate space	27 (60%)
Maternal comfort/Access to necessities	21 (48%)
Staff knowledge and support	14 (31%)
Maternal knowledge	12 (27%)
Other^b^	5 (11%)
**Support needed to increase KMC (n = 43)**^c^	
Educational material and training	35 (78%)
Adequate space	25 (58%)
Maternal comfort/Access to necessities	24 (53%)
Family and Staff support	7 (15%)
Monitoring equipment	1 (2%)

a 42/45 sites provided Kangaroo Mother Care.

b Financial (n = 2), distance to hospital (n = 2), equipment to facilitate monitoring/treatment of newborns while providing KMC (n = 1).

c Two sites reported no further support is needed.

### 3.3 Resources for managing healthcare-associated infections

On the survey day, 25 (56%) sites were caring for newborns with HAIs. Clinical sepsis was most common followed by gastrointestinal illness, meningitis, pneumonia, and skin/soft tissue infections. Among the 11 sites with microbiologic data available on the survey day, 8 (73%) HAIs were caused by Gram-negative organisms. During the previous year, 23 (51%) sites experienced at least one HAI cluster/outbreak, 14 (61%) of which were caused by Gram-negative pathogens. Ten sites were able to cohort affected newborns.

On the survey day, blood cultures, urine cultures, cerebrospinal fluid cultures, and susceptibility testing could be performed at 25 (56%), 24 (53%), 24 (53%) and 25 (56%) sites, respectively. All reported this was a typical day. Forty-four sites reported utilizing intravenous (IV) therapy; 22 (54%) had policies for IV dressing changes. Access to WHO-designated antimicrobial agents is shown (Supplement 2).

### 3.4 Water sources, hygiene, and cleaning/disinfection

*Water sources* used by sites included piped water (n = 39, 87%) and reservoirs or wells (n = 6, 13%). Thirty-one (69%) sites reported that the water supply to the hospital was treated. Boiled (35%), tap (35%), or bottled water (28%) were mainly used to prepare formula; one site had pre-made formula (2%). Distilled (42%), tap (42%), or bottled (16%) water was used to fill respiratory equipment (e.g., oxygen circuit). Water outages occurred ‘rarely’ at 28 (62%) and ‘daily’ at 7 (11%) sites.

*Hand hygiene* techniques were taught at 42 (93%) sites using posters, demonstrations, and lectures that described WHO recommendations for the “Five Moments for Hand Hygiene”. Nine (20%) sites did not teach the WHO recommendation to perform hand hygiene after touching patient surroundings. IP&C staff were available for the unit at 21 (47%) sites, and 11 (24%) formally monitored hand hygiene adherence. On the survey day, 35 (78%) sites reported available sinks in newborn care areas, but 7 (15%) reported fewer working sinks than usual. Four sites (9%) had no functioning sinks on the day of the survey. Most sites reported enough soap (n = 38, 84%), alcohol hand sanitizer (n = 44, 98%), and disposable towels (7/11, 64%). The virtual assessment demonstrated that 19 (42%) sites had barriers accessing sinks and 38 (84%) lacked one or more hand hygiene supplies (Table 2).

*Patient-care equipment*, including nasal cannula (n = 23/27, 85%), was often shared and/or reused (Table 4). Methods of cleaning/disinfecting patient care equipment and supplies varied (Table 4). Multi-dose medication vials were used at 35 (78%) sites.

**Table 4:** Shared and/or reused patient-care equipment and cleaning method.

**Equipment type**	**Shared and/or reused**^a^

Pulse oximeter	43/45 (96%)
Nasal cannula	23/27 (85%)
Thermometer	34/45 (76%)
Continuous Positive Airway Pressure	15/30 (50%)
Oxygen circuit	15/41 (36%)
Suction catheter	14/45 (31%)
Endotracheal tube	3/11 (27%)
Feeding tubes	8/44 (18%)
Syringes	4/45 (9%)

**Cleaning method**	**Number of sites (n = 44)**^b^

*Respiratory equipment*^c^	
Soap/water + bleach	16 (36%)
Chlorine	8 (18%)
Alcohol-based biocide	7 (16%)
Boiled water	5 (11%)
Soap/water only	3 (7%)
*Feeding tools*^d^	
Soap/water only	19 (43%)
Soap/water + disinfection	8 (18%)
Sterilize	8 (18%)
Water alone	3 (7%)
Family cleans	3 (7%)
N/A	3 (7%)
*Thermometer/Stethoscope/Pulse oximeter*	
Alcohol wipes	20 (45%)
Chlorine	18 (41%)
Soap/water only	6 (14%)

a n = positive response/N = equipment or supply available.

b No available response at one site.

c Respiratory equipment, e.g., oxygen circuit, endotracheal tube.

d Feeding tools, e.g., spoons, bottles, cups.

*Environmental cleaning and disinfection* were performed by an agency at 39 (87%) sites or by nurses and/or families at 6 (13%) sites. The types of cleaning agents and frequency of cleaning are shown (Table 5). During the virtual assessments, 20 (44%) sites had bedside clutter, 8 (18%) had inaccessible or overflowing rubbish cans, and 15/31 (48%) had unclean spaces in procedure areas (Table 2).

**Table 5:** Cleaning agents and frequency of cleaning for different environmental surfaces.

	Floors	Counters	Sinks	Beds
**Agent**				
Chlorine-based/bleach	38 (84%)	34 (75%)	31 (69%)	36 (80%)
Soap/water	4 (9%)	8 (18%)	12 (27%)	6 (13%)
Alcohol-based	2 (5%)	3 (7%)	2 (4%)	3 (7%)
**Adequate cleaning supplies available**				
Yes	35 (78%)	35 (78%)	32 (71%)	36 (80%)
No/Not sure	10 (22%)	10 (22%)	13 (29%)	9 (20%)
**Frequency**^a^				
Twice or more each day	29 (64%)	8 (18%)	1 (2%)	10 (22%)
Daily	13 (29%)	26 (58%)	23 (51%)	29 (64%)
Every other day	0 (0%)	3 (7%)	2 (4%)	0 (0%)
Weekly	1 (2%)	2 (4%)	1 (2%)	1 (2%)
As needed	6 (13%)	11 (24%)	12 (27%)	11 (24%)
At discharge	NA	NA	22 (49%)	NA

a Respondents able to select more than one option.

### 3.5 Personal protective equipment (PPE) and staff safety

PPE including sterile and non-sterile gloves were available at 24 (53%) sites on the survey day. Some sites implemented protective shoe coverings (n = 21, 47%), head coverings (n = 11, 24%), and changing to different scrubs/gowns when entering the unit (n = 25, 56%). During the virtual assessments, sharp containers were inaccessible or overflowing (n = 11, 24%), located on the floor (n = 24, 53%), and/or not secured on the counter (n = 14, 31%).

### 3.6 Respondents’ perceptions of newborn care needs

Themes elicited from respondents in open-ended questions included needs for space, equipment and infrastructure, nutrition, IP&C, staffing, and maternal and staff well-being (Table 6). Across many of these themes, respondents expressed concern for the experiences of mothers and stated that adequate support would encourage mothers to participate in KMC and reduce the risk of “contamination”.

**Table 6:** Themes and selected respondent quotes describing newborn care needs.

Theme	Selected quotes from respondents (n = number of comments regarding each specific theme)
**Space**	***Accommodate*** s***pecialpopulations****(n* = *19)* **“**We have a very small room. So if we have a bigger room with compartments for those babies who are fresh from maternity … born at home … we know that when you are born at home, probably it was not a clean and safe delivery.”
	***Accommodateequipment****(n* = *5)* **“**We also need another incubator, but if we don't have the space, it's difficult. We also need the phototherapy camera and the tunnel, but we don't know where to place it.” “We don't have a wider space, but if we did, we could use more of the radiant warmers more of the incubators.”
	***Mother-newborncare****(n* = *13)* “… maybe to expand the kangaroo [care] … We would love to keep the babies until they are 2000 g and not send them home when they are 1600 …” “… because it is very complicated for mothers when they do not have access to the room, they are forced to stay in the hallway and that is not a good way to treat a human.”
**Equipment &infrastructure**	***Respiratory****(n* = *29)* **“**My greatest need now is to be able to care for the extreme low-birthweight babies that reach us and I'm not asking for the moon, I'm just asking for simple basics. Can I get a CPAP there?” “More respiratory assistance equipment is needed including devices for non-invasive ventilation - a lot of babies are lost due to the fact that we can only provide oxygen therapy and further assistance cannot be provided if required.” **“**You know even pulse oximetry is not available because the sensor is not a disposable one, so the pulse oximetry is available, but the sensor is not there. “ “[…] because there is only one suction machine, which is used for several patients and the contamination is very high.”
	***Specific*** n***eeds****(n* = *35)* **“**The greatest need now is actually one thing. We need heaters, we need to be warm.” **“**We need incubators, we need KMC chairs.” **“**Phototherapy tunnel, because we have a lot of jaundice.” “Buy some supplies like gowns, and other medical supplies like gloves and will maybe use other lab apron.” “Central venous catheter, umbilical catheter, sterile fields.”
	***Storage****(n* = *6)* **“.**. I would like to have refrigerator for breast milk, separately for medication as well.” **“**And I want a locker for the mothers, because they are keeping everything outside in the corridor, the contamination is very high.”
**Nutrition**	***(n = 8)*** “Some of our babies if they stay for months, they are malnourished because we don't have nourishment for them.” “To provide more dietary support for the breastfeeding moms.” “I will realize my little dream of having a milk bank that is able to provide safe milk for newborns, especially those under 1000g whose mothers are either dead or in intensive care.”
**Infectionprevention &control**	***Education &systems****(n* = *24)* “Polices or guidelines like local develop guidelines for neonatal care and infection prevention policy … are some of the things that may be important for our state because we don't have policies in them.” “I believe that is the most important thing in infection prevention in our unit … regular training of our staff in infection prevention and control. And working on stewardship with the staff.” “There are many issues but the major one is the blood culture, urine basic culture, we don't have access to cultures so again we are guessing what bacteria we are treating we are throwing antibiotics that might not even be appropriate for the infection […].So I would also build a proper culture for the hospital.”
	***Environmentalfactors****(n* = *9)* **“**We would spend it on monthly running water. So the times when we run out of water it puts our patients at risk of infection.” **“**And more, just clean water, water to drink. The environment is very bad. Yeah, dining room for the mothers, who are just eating in the corridor.”
	***Equipmentre-use****(n* = *11)* **“**And maybe try and autoclave everything they use if it is possible. Because usually you come in, you do everything, you are careful their respiration, heart, and then they just have sepsis, it's hard.” “So the same sensor is used to different babies and that makes it so difficult sometimes managing the very tiny ones.” “I will spend it on equipment which are important for newborn care as you see we use most of materials by sharing with sick neonates.”
**Staffingissues**	***(n = 15)* “**And maybe the other thing is if I could have nurses, neonatal nurses trained because we don't have. If the people have proper training, it will impact on how they work.”
**Well-being**	***Mothers &******c******ommunities****(n* = *10)* “I would like to have shower for the mothers, and recreation, video for the mothers to watch while kangaroo mother care is done, I want the mothers to have tea and coffee in the middle of the night.” **“**So I think the main thing that we would stand to benefit is to make our kangaroo mother care bigger and to find some way to help mothers- to make their lives easier.” **“**The greatest need is to help the communities. That's a difficult thing to do.
	***Staff****(n* = *4)* **“**I want each physician to have his own stuff laundered every day, shoe to enter into the ICU, locker for every physician to put his clothes and things away from the neonatal ICU for the safety.” **“**Our nurses are ICU nurses they work 24/7 and almost all the time they are here somehow they miss refreshments, trainings, updates […] it's very heart wrenching they don't go out for lunch, they don't go out to pee, they don't go hangout with their friends because once they enter the ICU they have to stay very long hours in the evenings a lot.”

*Space:* Respondents identified the need for dedicated spaces for KMC, to meet maternal needs (e.g., a dining room, for preterm newborns, for isolation, and to accommodate equipment. One respondent noted, “*I cannot ventilate because I do not have the equipment. And even if I do, then I cannot put it into practice because I have a very, very tight space*.”

*Equipment, Supplies, and Infrastructure:* Respondents frequently cited the need for respiratory support including continuous positive airway pressure (CPAP) and pulse oximetry. One respondent reported having one CPAP machine for 40 beds, which lead to sharing and re-using supplies and equipment. Several described the need for PPE, e.g., gowns and gloves. Storage space for supplies and equipment and for storage of breastmilk and mothers’ belongings was another focus.

*Nutrition*: Some expressed the need for nutrition for infants and mothers. One respondent said, “We don't have breastmilk, we don't have parenteral nutrition for babies who stay on the ventilator for a long time. We rely on IV fluids. We cannot stop giving care, but we have no nutrition”.

*Infection Prevention & Control:* Many respondents highlighted the need for IP&C education and laboratory support including antimicrobial susceptibility testing. Others emphasized the need for clean water and avoiding reusing equipment to reduce risk of contamination. One respondent stated, *“We would spend [money] on monthly running water. The times when we run out of water, it puts our patients at risk of infection”.*

*Staff Training*: Respondents noted the need for neonatology education and training. One respondent said, “Just our major priority or the first thing we want to have is just getting a neonatologist or training manpower”.

*Well-being:* Respondents emphasized the need to improve the well-being of staff. They voiced that in addition to the training mentioned above, staff needed resources such as laundry, storage, and breaks. Respondents also highlighted needs such as water, food, restrooms, showers, and recreational materials for mothers. One respondent expressed “*I am sad each day when mothers struggle to go to use the restroom and they come back to stay with their babies and so I would change the setup to be more comfortable for moms*”.

## 4 Discussion

This multi-method study offers a unique look at IP&C practices and related resources available at diverse hospitals in Africa providing care to newborns. We found that most hospitals faced significant challenges addressing IP&C needs. These challenges included the need for additional space, equipment, supplies, education, training, and staff. Many of these challenges can only be addressed with substantial funding and extensive infrastructure improvements. However, data from studies such as this one are important as they vividly describe the extent of common challenges and provide priority areas for investment.

The countries represented by the hospitals in this study bear a disproportionate burden of HAIs, (Decraene et al., 2018) particularly caused by Gram-negative organisms. When microbiologic data were available, Gram-negative pathogens caused most HAIs and clusters/outbreaks. Notably, a minority of sites used the evidence-based practice of cohorting newborns during clusters/outbreaks to avoid additional transmission to other newborns (Hayward et al., 2022). Furthermore, many relatively narrow spectrum antibiotics used by the sites were consistent with WHO recommendations which may not reflect the susceptibility patterns seen in these countries (Richard et al., 2001). Thus, without consistent laboratory support for obtaining cultures and performing susceptibility testing to inform treatment and surveillance, HAIs caused by multidrug-resistant Gram-negative pathogens may be inadequately treated and under-estimated.

We also found that relatively low-cost IP&C practices could be further strengthened. While most sites taught the WHO's Five Moments for Hand Hygiene, 20% did not teach that hand hygiene should be performed after touching the patient care environment, an area that can be heavily contaminated by patient flora and serve as a reservoir for potential pathogens (FitzGerald et al., 2013; Bhalla et al., 2004).

Hand hygiene monitoring has been associated with an improved understanding of hand hygiene opportunities and increased adherence to hand hygiene practices, (Bhalla et al., 2004) but only a quarter of sites monitored hand hygiene. Many sites used alcohol-based hand sanitizers, but both the survey and video assessments revealed a lack of hand hygiene supplies and barriers to accessing sinks. Future work could develop strategies to support hand hygiene such as eliminating physical barriers to sink access, providing alcohol sanitizer at point of care, and monitoring hand hygiene adherence. IV dressing change policies, along with other preventive ‘bundle strategies’, have been associated with decreasing central line-associated bloodstream infections, (Huang et al., 2021) yet only half the sites that provided IV therapies had such policies for dressing changes. Providing window screens would both improve ventilation and reduce the risk of airborne and zoonotic infections (Ogoma et al., 2009).

Many sites had WASH practices that aligned with WHO recommendations to use chlorine-based/bleach products and perform a minimum of daily cleaning of environmental surfaces. However, the video assessments revealed crowding and clutter that could impede environmental cleaning suggesting that efforts to reducing crowding and clutter could improve the effectiveness of cleaning and disinfection. The U.S. Centers for Disease Control and Prevention have also increasingly emphasized safe water practices to reduce HAIs as municipal water can be contaminated by coliform bacteria and hospital pipes may harbor biofilms of potential bacteria and mycobacteria (Best Practices for Environmental Cleaning; Decraene et al., 2018; Hayward et al., 2022). Thus, sterile water is recommended to fill respiratory therapy equipment and prepare formula, but not all sites used sterilized water for these activities.

Additionally, IP&C strategies can be implemented to improve staff and visitor safety. For example, during the video assessments, sharps containers were frequently inaccessible, overflowing, and/or not secured on the wall. Focused attention to secure sharps containers and avoid overfilling could reduce the risk of sharps injuries to staff and potentially to visitors (Richard et al., 2001). Additionally, some PPE practices at the sites are not evidence-based such as wearing head or protective shoe coverings; disbanding these practices could be explored locally and ultimately be cost-savings.

KMC offers protection against HAIs likely due to reduced newborn contact with staff and improved breastfeeding (Arya et al., 2023). While passionate about performing KMC, respondents identified gaps in resources for KMC. Strengthening KMC through staff, mother, and community education and by improving maternal comfort through availability of water, food, and showers could expand the use of KMC. Many respondents emphasized addressing maternal needs as they noted that mothers who are unwell or frustrated cannot stay to look after their newborn or provide protection through KMC. Local work to reduce barriers to KMC due to incompatibility with cultural norms should also be considered (Best Practices for Environmental Cleaning).

While this study identified many shared experiences and challenges, the unique characteristics of each hospital including differing settings, resources, and specific needs means that a simple unifying solution to fit all does not exist. In this context, a more customized approach to address each care units’ IP&C challenges may prove more successful. The COVID era accelerated the use of virtual platforms, and we were able to review care settings from distances not possible just a few years ago. This medium facilitated our understanding of IP&C efforts and opportunities unique to each site. We anticipate that this method could be further developed to link remote hospitals with peers and with global experts who can address particular needs and provide tailored feedback.

## 5 Limitations

This study has several limitations. Respondents were selected because they were knowledgeable about the units providing care to small and/or sick newborns. This could have introduced potential biases including recall bias and socially desirable responses. Study sites were diverse; however, a larger cohort with more district hospitals may have offered further insights. Comparison between different types of hospitals was not done as many sites were unsure of their designation. While we routinely asked respondents if ‘this was a typical day’, we did not seek additional validation, e.g., alternative sources of census and staffing. Staffing for nursing was reported as total over 24 h and thus could not reliably calculate nurse to patient ratio per shift. Details regarding water treatment were not asked.

Due to limited internet connectivity in some areas, real-time virtual assessment of the sites could not always be performed. The virtual tour was planned in advance; thus sites could have made modifications prior to the tour. We did not ask the sites if they had made changes prior to the virtual assessment. Though there were separate questions in the survey to distinguish “shared” vs “reused” equipment, multiple respondents used the terms interchangeably. Thus we combined these responses in our analysis. Although we asked about the potential for cultural norms of families to be challenges to KMC, we did not explore staff cultural norms, which may also have been barriers to KMC. The qualitative results demonstrated the need to include questions about mother, community, and staff well-being in future studies.

## 6 Conclusions

This study highlights several opportunities for strengthening IP&C practices in hospitals caring for small and/or sick newborns. Results can be used by potential donors and health ministries for targeted areas of funding as well as by local neonatal units to inform their own practices and seek resources.

Respondents consistently identified the need for more space, equipment, personnel, supplies, education, and training for their units. These physical and workforce needs were not surprising and are costly. In addition, we identified economical strategies that largely involved strengthening implementation of evidence-based practices for hand hygiene, central line care, and WASH practices. To implement these strategies effectively further education and support are critical. Future work could explore creating a learning collaborative that promotes networking among hospitals and facilitates sharing practices, educational materials, ideas, and solutions (McMullan et al., 2013). Our findings suggest that virtual assessments are feasible and well accepted and could provide real-time assessment to hospitals by peers and/or global IP&C and environmental engineering experts. These approaches would offer opportunities to tailor IP&C strategies to the needs of individual hospitals.

## 7 PANCAS working group

Amanda Madison MD (Al Sabah Children's Hospital, Juba, South Sudan), Angela Okolo MD (Federal Medical Center, Asaba, Nigeria), Ashura Bakari MD MGCPS (Suntreso Government Hospital, Kumasi Ghana), Asrat Demtse MD (Black Lion Hospital, Addis Ababa, Ethiopia), Atnafu Mekonnen Tekleab MD MPH (St. Paul's Millennium Hospital, Addis Ababa Ethiopia), Bruktawit Aregawi MD (Adare General Hospital, Juba, South Sudan), Carine Judith Kiteze Nguinzanemou MD (Centre Hospitalier Universitaire Pédiatrique de Bangui, Central African Republic), Charlotte Ekoube Eposse MD (Hôpital Laquitinie Doualla, Cameroon), Chioma Ifeyinwa Obinna-Njoku MBBS FWACPaed (Federal Medical Center, Owerri, Nigeria), Crispen Ngwenya MBChB (Gweru Provincial Hospital, Zimbabwe), Cyprien Kouame Kouakou MD (Centre Hospitalier Universitaire Cocody, Abidjan, Côte d'Ivoire), Daouda Diamane Ndour MD (Centre Hospitalier National Dalal Jamm, Dakar, Senegal), Professeur Komi Deladem Azoumah (CHU Kara, Togo), Delania Lawrence MBChB (Rahima Moosa Mother and Children Hospital, South Africa), Dickens Onyango MD, Diomède Noukeu Njinkui MD (Hôpital Gynéco-obstétrique et pédiatrique de Douala, Cameroon), Éliane Kuissi Kamgaing MD (Université des Sciences de la Santé Libreville Gabon), Eric Ndihokubwayo MD (Hôpital Regional de Gitega, Burundi), Ezekiel Mupere MBChB MMed MS. PhD (Kawempe National Referral Hospital Makerere University, Kampala, Uganda), Ghislain Franck Houndjahoue MD (Centre Hospitalier Universitaire Pédiatrique de Bangui, Central African Republic), Granga D. Douna MBChB (CHU de la mere et de l'enfant, CHAD), Guillaume Ntawukuriryayo (‘hôpital de Ngozi, Burundi), Gwendoline L. Chimhini MBChB MMed MPH (Harare Children's Hospital, Zimbabwe), Hailu Berta Deregh MD (Queen Zewditu Memorial Hospital, Ethiopia), Hilda Angela Mujuru MBCHB MMED Paeds Msc (Harare Children's Hospital, Zimbabwe), Himels Bonsa Tessema MD (Dilla General Hospital, Ethiopia), Ibrahim Mark Kapuwa MD (Kenema Government Hospital University of Sierra Leone Teaching Hospitals, Sierra Leone), Irene Chebet MD (Soroti Regional Referral Hospital, Uganda), Jacqueline Gyapomaa Asibey MBcHB (Holy Family Hospital-Techiman, Ghana), Jean Bosco Habonimana (Hospital de District de Muyinga, Burundi), Jean Chrysostome Gody MD (Centre Hospitalier Universitaire Pédiatrique de Bangui, Central African Republic), Joy Fredericks MBBCh (Rahima Moosa Mother and Children Hospital, South Africa), Justin Bruno Tongun MBChB PhD (Al Sabah Children's Hospital, Juba, South Sudan), Lala N'drainy Sidibe MD (CHU Gabriel, Touré, Mali), Lawrence Waguma RN (Muhoroni County Hospital, Kenya), Lloyd Tooke MBBS (Groote Schur Hospital, Cape Town, South Africa), Mary Nyanzi Mbchb Mmed (Kawempe National Referral Hospital Makerere University, Uganda), Millicent Oloo RN (Kisumu County Referral Hospital, Kenya),Mohammedyassin Redi Awel MD (Wilkite University Hospital, Ethiopia), Naana A. Wireko Brobby MBChB (Komfo Anokye Teaching Hospital, Ghana), Nellie V. T. Bell MD DCH (Ola During Hospital Sierra Leone), Nicole Tchiakpe MD (Centre hospitalier et Universitaire de la mere et de l'enfant, Lagune, Benin), Rafaella Marino MD (Centre Hospitalier Universitaire Pédiatrique de Bangui, Central African Republic), Ranita Luke MBChB (Ola During Hospital, Sierra Leone), Salim Abdallah Nouzhat (Moroni hospital, Moroni, Comoros), Samrawit Russom Weldeselliassie MD (Menelik II comprehensive specialized Hospital, Ethiopia), Scholastica Mzera RN (Muhoroni County hospital, Kenya) Synthia Ningatoloum Nazita MD (Centre Hospitalier Universitaire Communautaire de Bangui, Central African Republic), Victoria Nakibuuka Kirabira MD (St Francis Nsambya Hospital, Kampala, Uganda), Workeabeba Abebe MD MPH (Black Lion Hospital, Addis Ababa, Ethiopia), Yenealem Ayele Yitayeh MD (Yekatit 12 Hospital, Addis Ababa, Ethiopia), Yolande Puepi Djike MD (Hopital Regional de Buea, Cameroon), Yousif Zubair MD (Juba Teaching Hospital, South Sudan).

## Funding

10.13039/100000865Bill and Melinda Gates Foundation grant INV-002005.

Irene Frantzis supported by NIAID T32 AI007531.

Julia Johnson supported by NIH K23HD100594.

## CRediT authorship contribution statement

**Irene Frantzis:** Writing – original draft, Project administration, Methodology, Investigation, Formal analysis, Data curation. **Stéphanie Levasseur:** Writing – review & editing, Supervision, Data curation. **Jack Huebner:** Writing – review & editing, Data curation. **Maitry Mahida:** Project administration. **Philip Larussa:** Writing – review & editing, Supervision, Conceptualization. **Wilmot James:** Supervision, Conceptualization. **Workeabeba Abebe:** Writing – review & editing, Methodology, Formal analysis, Data curation. **Crispen Ngwenya:** Writing – review & editing, Investigation, Data curation. **Ezekiel Mupere:** Writing – review & editing, Methodology, Data curation. **Susan L. Rosenthal:** Writing – review & editing, Supervision, Formal analysis. **Janna Patterson:** Writing – review & editing, Methodology, Investigation. **Julia Johnson:** Writing – review & editing, Methodology, Investigation. **Renate Strehlau:** Writing – review & editing, Data curation. **Sileshi Lulseged:** Project administration, Data curation. **Lawrence R. Stanberry:** Writing – review & editing, Supervision, Resources, Methodology, Investigation, Funding acquisition, Formal analysis, Conceptualization. **Lisa Saiman:** Writing – review & editing, Visualization, Supervision, Resources, Methodology, Investigation, Funding acquisition, Formal analysis, Data curation, Conceptualization.
