# Supplementary material for: Infection prevention and control and related practices in African neonatal units: The Pan-African neonatal care assessment study (PANCAS)
Source: Int J Hyg Environ Health. 2024 Jun;259:114357. doi: 10.1016/j.ijheh.2024.114357 (PMC11163474; doi:10.1016/j.ijheh.2024.114357)
Supplement: Multimedia component 1 [file mmc1.doc]

**Supplement 1**

PANCAS Survey

*Thank you for participating in our survey. Our study team from Columbia University is interested in better understanding African facilities that care for inborn small or sick newborns less than 30 days of age. We are particularly interested in potential factors related to infection prevention and control that put newborns at risk for healthcare-associated (nosocomial) infections.*

*We will be asking questions about how newborns are cared for. Most questions will focus on the unit today, but some questions are about the impact of the COVID-19 pandemic. We will record our conversation to ensure that we accurately capture your responses. After our study team reviews the recording, we will delete it.*

***Please review the survey prior to our call. There may be some questions that you are not sure about and you can discuss with other members of your team before our call. For example, we have yellow-highlighted Questions 102-117 – see below.***

*We would like to do the survey with you after your rounds, if possible. It may help you answer the questions if you have a list of the inborn small or sick newborns currently in your unit. We will not ask you about individual patients or individual staff or collect any patient or staff identifiers.*

*The survey will be followed by a virtual tour of the unit where newborns receive care. We will videotape the tour so we can review the tour to be sure we accurately describe the unit. After we review the videotape, we will delete it.*

*Do you have any questions before we begin?*

**Identification of Study Site**

Name of institution:

Location (City/ Province/ Region/ Country): ____________________

**Type of facility**

What is your facility’s designation? *Provide the most applicable responses*

- National
- Regional
- District
- Academic
- Other:

**Is your facility:** *Provide the most applicable responses*

- - Public (government)
- Private
- Public/Private partnership
- Missionary hospital


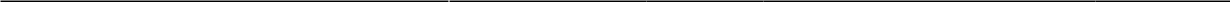


**Clinical Facilities/ Types of patients**

*We will begin by asking some questions about newborns < 30 days of age including the space and unit where they are cared for. We will primarily focus on the small and sick newborns who were born at your facility, but ask some questions about newborns born outside your facility, if relevant.*

1. Can you please describe where small or sick newborns less than 1 month of age born at your facility are cared for? ____________________________________________________________________
2. Does this space also hold small or sick newborns born outside your facility (“born prior to admission”) or readmitted after being discharged from your facility?
   - 1. Yes; *ask respondent to elaborate* _________________________________________________
     2. No; only small or sick newborns born at this facility
3. Do you accept small or sick newborns born in other hospitals or born at home? *Provide responses*
   - 1. Yes, other hospitals
     2. No, other hospitals
     3. Yes, born home
     4. No, born home
4. Are small or sick newborns born in other hospitals or born at home co-mingled with inborn small or sick newborns?
   - 1. Yes
     2. No
     3. Not applicable
5. How many cribs (bassinets)/ beds**/ isolettes (incubators)/ radiant warmers/ phototherapy beds are available today to care for small or sick newborns?
   - 1. Cribs/ bassinets: __________
     2. Beds: __________
     3. Isolettes/ incubators: ________
     4. Radiant warmers: ________
     5. Phototherapy beds: _______
   - *including shared bed with mother*
6. Is today a typical day?
   - 1. Yes
     2. No
     3. If no, why?
7. How many small or sick newborns are you caring for today?
   - 1. Number (including zero):
     2. Not applicable (no small or sick newborns today)

1. How many small or sick newborns would you typically be caring for prior to the COVID pandemic?
   1. Number (including zero):
2. How many cribs (bassinets)/ beds/ isolettes (incubators)/ radiant warmers/ phototherapy beds have more than one newborn today?
   1. Number (including zero):
   2. Not applicable (no small or sick newborns today)
3. Do you care for newborns whose birthweight was under 1500 grams?
   1. Yes
   2. No
4. If yes, how many newborns with birthweight under 1500 grams are in your unit today?
   1. Number (including zero):
   2. Not applicable (don’t care for newborns birthweight < 1500 grams)
5. Are infants more than 1 month old co-mingled with small or sick newborns?
   1. Yes
   2. No
6. How many infants more than 1 month of age are you caring for today?
   1. Number (including zero):
   2. Not applicable (don’t comingle)
7. What are common barriers to timely discharge for small or sick newborns from your unit? *All that apply*
   1. Social issues
   2. Lack of transportation
   3. Parental preference
   4. Lack of adequate space in a lower-acuity unit
   5. Other:________________________________________
   6. None

**Impact of COVID-19**

*Now we would like to ask you a few questions about the impact of COVID-19 on your facility.*

1. Can you describe if/ how you evaluate mothers for COVID-19? Are they tested or screened for symptoms? _______________________________

***If no screening/ testing, please answer ‘Not applicable’ questions 16-19 & go to question 20***

1. What is your policy for small or sick newborns born to mothers with confirmed or suspected COVID-19? *For example, do you:*
   1. Separate newborn from mother
   2. Keep newborns with mother on isolation
   3. Keep newborns with mother without any isolation
   4. Other:
   5. Not applicable
2. Are mothers with confirmed or suspected COVID-19 allowed to breastfeed small or sick newborns?
   1. Yes
   2. No
   3. Not applicable
3. How many small or sick newborns are you caring for today born to mothers with confirmed or suspected COVID-19?
   1. Number (including zero):
   2. Not applicable
4. How many small or sick newborns are you caring for today with confirmed or suspected COVID-19?

a. Number (including zero): __

- 1. Not applicable

1. Since the COVID-19 pandemic, has the number of admissions of small or sick newborns:
   1. Increased
   2. Decreased
   3. Remained the same
2. Have you seen an increase in admissions of small or sick newborns born at home since the COVID-19 pandemic started?
   1. Yes
   2. No

**Visitor Practices**

*Now we will ask some questions about visitors.*

- 1. Can you please describe your visitor practices for small or sick newborns? *Ask about screening for illnesses among visitors, number of visitors at the bedside, types of visitors allowed*
  2. Have your visitor practices changed due to COVID-19?
     1. Yes; *prompt respondent to answer how so*
     2. No
  3. What types of care do mothers provide to their small or sick newborns?
  4. What types of care do visitors other than mothers provide for small or sick newborns?
  5. What types of care do visitors provide for another family’s small or sick newborn?
  6. Can more than one person visit a small or sick newborn at the same time?
     1. Yes
     2. No
  7. Can children younger than 12 years old visit small or sick newborns?
     1. Yes
     2. No

1. How many visitors did you see in the unit today?
   - 1. Number (including zero):
     2. Unsure
   1. How many visitors do you typically have each day?
      1. Number:
      2. Unsure

**Kangaroo Care**

*The following questions are about Kangaroo care.*

1. Can you please describe how kangaroo care is done for small or sick newborns in your unit? *Obtain information about how kangaroo care is initiated, address skin to skin contact, recommended duration of Kangaroo care, and breastfeeding. ______________________________*

***If Kangaroo care unavailable, answer ‘Not applicable’ for questions 32-38 and go to question 39***

1. Have Kangaroo care practices changed due to COVID-19? *Information about influences from hospital, mother, family, staff, more, fewer, different strategies, space issues, etc.*
2. How many small or sick infants are receiving Kangaroo care today?
   1. Number (including zero):
   2. Not applicable (Kangaroo care not performed at facility)
3. Is today a typical day?
   1. Yes
   2. No
   3. Not applicable (Kangaroo care not performed at facility)
4. If resources were available, how many additional small or sick newborns would be eligible for Kangaroo care today?
   1. Number (including 0): _____________________
   2. Not applicable (Kangaroo care not performed at facility)
5. Where do mothers provide Kangaroo care?
   1. At bedside
   2. Separate area
   3. Other (specify):
   4. Not applicable (Kangaroo care not performed at facility)
6. Do mothers providing Kangaroo care have access to the following? *All that apply*
   1. Bed
   2. Chair
   3. Food
   4. Water
   5. Restroom
   6. Not applicable (Kangaroo care not performed at facility)
7. What are the eligibility criteria for small or sick newborns to receive Kangaroo care?
   1. Criteria *(include preterm/ term, weight cutoff, medical stability): ________________*
   2. Not applicable (Kangaroo care not performed at facility)

- 1. Do you think your healthcare team believes in the benefits of Kangaroo Care?
     1. Yes
     2. No
     3. Unsure
  2. Do you think that mothers believe in the benefits of Kangaroo Care?
     1. Yes
     2. No
     3. Unsure
  3. What educational resources are available for Kangaroo Care for your healthcare team? __________
  4. Are there educational resources for Kangaroo Care available for mothers?
     1. Yes; *Please describe*:
     2. No
  5. Do you think that your healthcare team is able to provide enough support to mothers to provide Kangaroo care?
     1. Yes
     2. No; *Please explain: __________________________________________*
  6. Do you think that families provide enough support to mothers to provide Kangaroo care?
     1. Yes
     2. No; *Please explain): __________________________________________*
  7. What are the challenges you face to providing Kangaroo Care? *For example:*
     1. Mothers’ medical condition
     2. Small or sick newborn’s medical condition (perceived as too fragile or sick)
     3. Incompatibility with cultural norms/ traditional practices
     4. Healthcare worker familiarity with kangaroo care
     5. Mother’s familiarity with kangaroo care
     6. Family support to mothers
     7. Staff support to mothers
     8. Access to food/washing/toileting for mothers
     9. Childcare support for older siblings
     10. Adequate space
     11. Other:

1. Is there something that you think could increase Kangaroo care in your unit? _______

**IV Use and Administration of IV Fluids**

*The following questions will be about administering intravenous fluids*

1. Does your hospital routinely use intravenous (IV) fluids for small or sick newborns?
   1. Yes
   2. No

***If no IV fluids used, answer not applicable for questions 48-52 and go to question 53.***

1. If yes, what type of IVs do you use?
   1. Peripheral
   2. Central
   3. Both
   4. Not applicable, IV fluids not used.
2. Are any small or sick newborns receiving IV fluids today?
   1. Yes
   2. No
   3. Not applicable, IV fluids not used.
3. If yes, how many small or sick newborns are receiving IV fluids today?
   1. Number (including 0):
   2. Not applicable, IV fluids not used.
4. Do you have a rule about how often IV dressings are changed?
   1. Yes
   2. No
   3. Not applicable, IV fluids not used.
5. If yes, what is it? _________________________________________________
   1. Not applicable, IV fluids not used.

**Healthcare-associated Infections/ Antibiotics/ Laboratory Practices** *Next we will ask questions about your laboratory and antibiotic use.*

1. Are bacterial cultures on blood, urine or CSF available today? *All that apply*
   1. Yes; Blood
   2. Yes; Urine
   3. Yes; CSF
   4. None
   5. Unsure
2. Are bacterial cultures on blood, urine or CSF typically available? *All that apply*
   1. Yes; Blood
   2. Yes; Urine
   3. Yes; CSF
   4. No
3. If yes, can the lab provide antibiotic sensitivities today?
   1. Yes
   2. No
   3. Unsure
4. Is the lab typically able to provide antibiotic sensitivities?
   1. Yes
   2. No
   3. Not applicable, if sensitivities unavailable.
5. How many small or sick newborns are being treated now for a nosocomial infection, meaning they developed infection three or more days after they were admitted to your unit?
   1. Number (including zero):
   2. Information not available
6. What types of nosocomial infections do they have and how many small or sick newborns have each type?
   1. Gram negative bacteria (number: )
   2. Gram positive bacteria (number: )
   3. Gastrointestinal illness (number: )
   4. Skin and soft tissue infections (number: )
   5. Clinical sepsis, no organism identified (number: )
   6. Pneumonia (number: )
   7. Fungal (number:____)
7. None
8. Information not available

1. Over the past year, has your unit had a cluster or outbreak that involved three or more small or sick newborns with the same type of infection within a week?
   1. Yes
   2. No
   3. Information not available
2. If yes, what type of outbreak occurred that involved small or sick newborns? *All that apply*
   1. Gram negative bacterial outbreak
   2. Gram positive bacterial outbreak
   3. Gastrointestinal illness
   4. Skin and soft tissue infections
   5. Clinical sepsis, no organism identified
   6. Information not available
3. If yes, what was the source of the outbreak?
   1. Source(s) if known:
   2. Not applicable, no outbreaks identified.
4. Do you cohort small and sick newborns based on the outbreak/ type of infection?
   1. Yes
   2. No
   3. Not applicable, no outbreaks identified.
5. What is your standard empiric antibiotic therapy for small or sick newborns with a new infection (after day 3 of life)?
6. Where are antibiotics obtained?
   1. Your hospital pharmacy
   2. Community pharmacy (purchased outside the hospital for inpatient use)
   3. Both

1. Which of the following antibiotics do you have available in your facility today? *All that apply*
   1. Ampicillin
   2. Gentamicin
   3. Cefotaxime
   4. Vancomycin
   5. Benzylpenicillin
   6. Cloxacillin
   7. Amikacin
   8. Piperacillin/ tazobactam
   9. Meropenem
   10. Other:
   11. None

**Staffing/ Training**

*I will ask questions about staffing in your unit.*

1. In the past 24 hours, how many nurses were working in your unit?
   1. Number:
2. On a typical day, about how many nurses work in your unit over 24 hours?
   1. Number:
3. In the past 24 hours, how many nurses from other units were assigned to your unit?
   1. Number (including zero):
4. On a typical day, about how many nurses from other units are assigned to your unit over 24 hours?
   1. Number:
5. In the past 24 hours, how many doctors were working in your unit?
   1. Number:
6. On a typical day, about how many doctors work in your unit over 24 hours?
   1. Number:
7. Of the doctors working today, how many have completed formal post-graduate pediatrics training?
   1. Number:
8. Can you describe the type of post graduate pediatrics training they received?

a.

b. Not applicable

1. Of the doctors working today, how many completed formal post-graduate neonatology training? a. Number:
2. Can you please describe the type of post graduate neonatology training they received?

a.

b. Not applicable

**Hand Hygiene/ Infection Prevention and Control (IP&C)**

*Now we will ask some questions about hand hygiene/ IP&C measures.*

- 1. What are the practices for healthcare workers (HCWs) entering the unit? *(e.g., washing/ scrubbing at the entrance, bare below the elbows, wearing scrubs, wearing head or shoe coverings): __________*
  2. Can you describe how HCWs caring for small or sick newborns are taught to do hand hygiene?
     1. Posters
     2. Demonstrations
     3. Lectures
     4. Other:
  3. When are HCWs expected to perform hand hygiene? *All that apply*
     1. Before touching the patient
     2. Before clean/ aseptic procedures
     3. After body fluid exposure/risk
     4. After touching a patient
     5. After touching patient surroundings
     6. Other:
  4. Does your hospital have a formal standard of practice to monitor adherence to hand hygiene on units where small or sick newborns are cared for?
     1. Yes
     2. No

1. Can you describe how adherence is monitored on units where small or sick newborns are cared for? a. __________________________________________________________________________
   - 1. Not applicable, adherence is not monitored

1. If yes, how frequently?
   1. Weekly
   2. Monthly
   3. Quarterly
   4. Annually
   5. No specific frequency
   6. Not applicable, adherence is not monitored
2. Are the data for hand hygiene adherence provided back to the unit staff?
   1. Yes
   2. No
   3. Not applicable, adherence is not monitored
3. Do you have IP&C staff that covers this unit?
   1. Yes; (explain)______________________________
   2. No
4. Is there at least one sink available for handwashing in each area where small or sick newborns are cared for?
   1. Yes
   2. No
5. How many sinks are working today?
   1. Number (including zero):
6. How many sinks are typically working?
   1. Number (including zero):
7. Do you have enough soap today?
   1. Yes
   2. No
8. Do you typically have enough soap available?
   1. Yes
   2. No
9. Do you have enough alcohol sanitizer for hand hygiene today?
   1. Yes
   2. No

1. Do you typically have enough alcohol sanitizer for hand hygiene?
   1. Yes
   2. No
2. What do you use in your unit to dry hands?
3. Do you use disposable towels?
   1. Yes
   2. No
4. Do you have enough disposable towels today?
   1. Yes
   2. No
   3. Not applicable, don’t use disposable towels
5. Do you typically have enough disposable towels?
   1. Yes
   2. No
   3. Not applicable, don’t use disposable towels

**Water Source**

*Now I will ask some questions about water source in your unit.*

1. What is the water source for your unit’s hand hygiene (*for example, is it piped, from a well, carried to the unit, etc.*)? ________________________________________________________
2. Is the water treated?
   1. Yes
   2. No
   3. Unsure
3. What water do you use to mix formula for your small or sick newborn? ________________________
4. Where are feeds stored for your small or sick newborns? *(e.g., refrigerator for expressed breastmilk, storage unit for formula*) _______________________________
5. What water supply is used for respiratory equipment such as high flow cannulae or CPAP? ________

1. How often do you have water outages?
   1. Daily
   2. Weekly
   3. Monthly
   4. Rarely

**Environment and Equipment Cleaning**

*The following questions will be about cleaning routines in your unit. If you aren’t certain about these answers, it’s okay. Please ask a team member who will know the answer.*

- 1. Can you please describe who routinely cleans the unit? *For example, is there a specific agency, do they also clean other units? ___________________________________________________________*
  2. How often are the floors cleaned? *Select all that apply*
     1. Every day
     2. Twice each day
     3. Every other day
     4. Other routine frequency: specify:
     5. As needed

1. What cleaning agent is used to clean the floors?
   - 1. Specify:__________________________
   1. Do you typically have enough of this agent to clean the floors?
      1. Yes
      2. No
      3. If not, what do you use?
   2. How often are the counters cleaned? *Select all that apply*
      1. Every day
      2. Twice each day
      3. Every other day
      4. Other routine frequency: specify:
      5. As needed
   3. What cleaning agent is used to clean the counters?
      1. Specify:
   4. Do you typically have enough of this agent to clean the counters?
      - 1. Yes
        2. No
        3. If not, what do you use?

1. How often are the cribs (bassinets) / beds/ isolettes (incubators)/ radiant warmers/ phototherapy beds cleaned? *Select all that apply*
   1. Every day
   2. Twice each day
   3. Every other day
   4. After patient discharge
   5. Other routine frequency: specify:
   6. As needed
2. What cleaning agent is used to clean the cribs/ beds/ isolettes (incubators)/ radiant warmers/ phototherapy beds?
   1. Specify:
3. Do you typically have enough of this agent to clean the cribs/ beds/ isolettes (incubators)/ radiant warmers/ phototherapy beds?
   - 1. Yes
     2. No
     3. If not, what do you use?
4. How often are the sinks cleaned? *Select all that apply*
   1. Every day
   2. Twice each day
   3. Every other day
   4. Other routine frequency: specify:
   5. As needed
5. What agent is used to clean the sinks?
   1. Specify:
6. Do you typically have enough of this agent to clean the sinks?
   1. Yes
   2. No
   3. If not, what do you use?
7. How often are the bed linens changed? *Select all that apply*
   1. Every other day
   2. After patient discharge
   3. Other routine frequency: specify:
   4. As needed
8. Are there sufficient quantities of linens to maintain your routine linen changing protocol?
   1. Yes
   2. No

1. Who provides the crib (bassinet)/bed/ isolette (incubator)/ radiant warmer/ phototherapy bed linen?
   1. Hospital laundry
   2. The family
   3. Both

**Patient Care Equipment**

*Now we would like to discuss patient care equipment with you.*

1. What respiratory equipment do you use? *E.g., CPAP, high flow nasal cannulae, ventilators*
   1. Specify:
2. How do you clean the respiratory equipment?
   1. Specify:
3. How do you clean bottles/ spoons/ feeding tools?
   1. Specify:
4. What medical equipment is shared between small or sick newborns?
   1. CPAP
   2. Thermometers
   3. Pulse oximeter
   4. Nasal cannulae
   5. Suction catheters
   6. Other:______________________________________________
5. What medical equipment do you reuse? *For example*
   1. Endotracheal tube
   2. Syringes
   3. Ventilator circuit
   4. Sterile gloves
   5. Feeding tubes
   6. Nasal cannulae
   7. Pulse oximeter
   8. Thermometers
   9. Suction catheters
   10. Other:
6. How do you clean equipment that is reused?

**Supplies**

*Now we will ask questions about supplies in your unit.*

1. Do you use multi-dose vials to give medications to more than one small or sick newborn?
   1. Yes
   2. No
2. If yes, how frequently?
   1. Most days
   2. 1-2 times each week
   3. Once a month
   4. Never
3. What type of gloves are available in your unit? *All that apply*.
   1. Sterile gloves
   2. Re-processed sterile gloves
   3. Non-sterile gloves
   4. None of above
4. Do you have enough sterile gloves for today?
   1. Yes
   2. No
   3. Not applicable, sterile gloves aren’t used
5. Do you typically have enough sterile gloves?
   1. Yes
   2. No
   3. Not applicable, sterile gloves aren’t used
6. Are sterile gloves reused when caring for the same small or sick newborn?
   1. Yes
   2. No
   3. Not applicable, sterile gloves aren’t used
7. Are sterile gloves reused when caring for different small or sick newborns?
   1. Yes
   2. No
   3. Not applicable, sterile gloves aren’t used
8. Do you have enough non-sterile gloves for today?
   1. Yes
   2. No
   3. Not applicable, non-sterile gloves aren’t used

1. Do you typically have enough non-sterile gloves?
   1. Yes
   2. No
   3. Not applicable, non-sterile gloves aren’t used
2. Are non-sterile gloves reused when caring for the same small or sick newborn?
   1. Yes
   2. No
   3. Not applicable, non-sterile gloves aren’t used
3. Are non-sterile gloves reused when caring for different small or sick newborns?
   1. Yes
   2. No
   3. Not applicable, non-sterile gloves aren’t used
4. Do you use gowns?
   1. Yes
   2. No
5. What type of gowns do you have? *All that apply.*
   1. Cloth
   2. Disposable
   3. Not applicable, gowns aren’t used
6. Do you have enough gowns for today?
   1. Yes
   2. No
   3. Not applicable, gowns aren’t used
7. Do you typically have enough gowns?
   1. Yes
   2. No
   3. Not applicable, gowns aren’t used
8. Are cloth gowns reused before being laundered?
   1. Yes
   2. No
   3. Not applicable, cloth gowns aren’t used

1. Are disposable gowns reused when caring for different newborns?
   1. Yes
   2. No
   3. Not applicable, disposable gowns aren’t used
2. Do you use aprons?
   1. Yes
   2. No
   3. Not applicable, aprons aren’t used
3. Do you have enough aprons for today?
   1. Yes
   2. No
   3. Not applicable, aprons aren’t used
4. Do you typically have enough aprons?
   1. Yes
   2. No
   3. Not applicable, aprons aren’t used
5. Are aprons reused when caring for different newborns?
   1. Yes
   2. No
   3. Not applicable, aprons aren’t used

**Funding wish list**

1. If you had money for your unit, what will you spend it on?
2. What do you think is your greatest need?
