# Supplementary material for: Infection prevention and control and related practices in African neonatal units: The Pan-African neonatal care assessment study (PANCAS)
Source: Int J Hyg Environ Health. 2024 Jun;259:114357. doi: 10.1016/j.ijheh.2024.114357 (PMC11163474; doi:10.1016/j.ijheh.2024.114357)
Supplement: Multimedia component 2 [file mmc2.docx]

**Supplement 2**

Antibiotic Availability and Pharmacy Source

| Available Antibiotics on day of Survey | |
| --- | --- |
| Antibiotic | Number of sites where available |
| Ampicillin | 39 (87%) |
| Gentamicin | 39 (87%) |
| Cefotaxime | 37 (82%) |
| Benzylpenicillin | 24 (53%) |
| Meropenem | 13 (29%) |
| Imipenem | 5 (11%) |
| Amikacin | 17 (38%) |
| Vancomycin | 18 (40%) |
| Cloxacillin | 22 (49%) |
| Piperacillin/tazobactam | 5 (11%) |
| Ceftriaxone | 8 (18%) |
| Ciprofloxacin | 7 (16%) |
| Linezolid | 1 (2%) |
| Ceftazidime | 2 (4%) |
| Metronidazole | 5 (11%) |
| Source of Antibiotics | |
| Hospital pharmacy | 16 (36%) |
| Community pharmacy | 3 (7%) |
| Both hospital and community | 26 (58%) |
